# A 6-year audit of public-sector MR utilisation in the Western Cape province of South Africa

**DOI:** 10.4102/sajr.v26i1.2464

**Published:** 2022-07-22

**Authors:** Yusuf Parak, Razaan Davis, Michelle Barnard, Amanda Fernandez, Keith Cloete, Matodzi Mukosi, Richard D. Pitcher

**Affiliations:** 1 Division of Radiodiagnosis, Department of Medical Imaging and Clinical Oncology, Faculty of Medicine and Health Sciences, Stellenbosch University, Cape Town, South Africa; 2 Department of Radiodiagnosis, Tygerberg Hospital, Cape Town, South Africa; 3 Sub-Directorate Medical Imaging Services, Directorate: Health Technology, Western Cape Government Health and Wellness, Cape Town, South Africa; 4 Western Cape Government Health and Wellness, Cape Town, South Africa; 5 Tygerberg Hospital, Cape Town, South Africa

**Keywords:** radiology, public sector, middle income country, magnetic resonance imaging (MRI), utilisation, equitable access, health equity

## Abstract

**Background:**

Disparities in MR access between different countries and healthcare systems are well documented. Determinants of unequal access within the same healthcare system and geographical region are poorly understood.

**Objective:**

An analysis of public sector MR utilisation in South Africa’s Western Cape province (WCP).

**Methods:**

A retrospective study of WCP MR and population data for 2013 and 2018. MR units/10^6^ people, studies, and studies/10^3^ people were calculated for each year, for the whole province and the ‘western’ and ‘eastern’ referral pathways, stratified by age (0–14 years, > 14 years)

**Results:**

Between 2013 and 2018, the WCP population increased 8% (4.63 vs 5.08 × 10^6^ people) while MR resources were unchanged (‘western’ = 2 units; ‘eastern’ = 1), equating to decreasing access (units/10^6^ people) for the province (0.65 vs 0.59; –9.2%), the ‘western’ (0.97 vs 0.9; –7.2%) and ‘eastern’ (0.39 vs 0.35; -10.3%) pathways. In 2013, 40% (4005/10 090) of studies were in the ‘eastern’ pathway serving 55% (2 066 079/4 629 051) of the population. Between 2013 and 2018 ‘eastern’ population growth (*n* = 286 781) exceeded ‘western’ (*n* = 168 469) by 70% (*n* = 118 312). By 2018, 38% (7939/12 848) of studies were performed in the ‘eastern’ pathway, then serving 56% (2 849 753/5 084 301) of the population. Among 0–14-year-olds, ‘western’ utilisation (studies/103 people) exceeded ‘eastern’ by a factor of approximately 2.4 throughout. In patients > 14 years, the utilisation differential increased from 1.78 to 1.98 in the review period.

**Conclusion:**

Ensuring equitable services on the same healthcare platform requires ongoing surveillance of resource and population distribution. MR access can serve as a proxy for equity in highly specialised services.

## Introduction

Technological advances, together with population growth, increased life expectancy, the evolving burden of disease and defensive medical practice have led to a global increase in both the demand for, and the utilisation of, diagnostic imaging services. This is particularly true for MR.^1,2^ The modality’s unsurpassed soft tissue contrast, sensitivity, specificity, as well as the absence of ionising radiation, contribute to ever-expanding clinical indications.^1,3,4^ MR utilisation in the United States (US) and Canada showed annual average increases of 5% and 10%, respectively, between 2003 and 2019.^5^ The United Kingdom (UK) usage increased by an average of 10% per year from 2012 to 2019, and in 2018 alone, 3.7 million investigations were performed nationally.^6^ In comparison, public sector MR utilisation in the Western Cape province (WCP) of South Africa (SA) increased by an average of 3% annually between 2009 and 2019.^7^ However, MR is a capital- and labour-intensive modality and burgeoning clinical utilisation has the potential to increase healthcare costs substantially.^8,9^ Furthermore, increasing imaging costs can accentuate existing inequalities in global access to healthcare, by widening the service gap between well- and poorly resourced environments.

The optimal number of MR scanners per million people has not been established.^10^ Japan, the US, the Organization for Economic Cooperation and Development (OECD) countries, and the UK have 55, 40, 17 and 7 units/million people respectively.^5,11^ The BRICS nations such as Brazil, Russia, India and China have 6.8, 5.1, 0.6 and between 1.6 and 4.45 units/million, respectively.^12,13,14,15^ By comparison, the South African, Zimbabwean, Nigerian, Zambian and Tanzanian public healthcare sectors have 0.33, 0.2, 0.11, 0.06 and 0.05 scanners/million people, respectively.^1,16,17,18,19^

There is also wide global variation in the clinical utilisation of MR. For example, Germany, the US, Japan, Canada and the UK perform 131, 118, 112, 56 and 53 scans per 1000 citizens per year, respectively.^5^ The WCP performed 2.5 scans per 1000 people in 2019.^7^ There are also marked global disparities in the annual clinical workload of MR units. In 2019 MR scanners in Turkey, UK and Canada performed an average of 5993, 8025 and 9872 studies, respectively,^5,20^ while WCP units performed an average of 4168 scans in 2017.^21^ There also exists substantial variations in the distribution and utilisation of resources within countries. MR scanners are typically concentrated in the urban areas. This is particularly true in resource constrained environments, such as Southern Africa, where units are confined to the largest cities.^1,2,7,16,19,21^ Additionally, in the US, persons who are 65 years and older undergo three times more MR investigations than their younger counterparts.^8^

Equitable access to essential health services is a key consideration in the United Nations (UN) 2030 Agenda for Sustainable Development and its Sustainable Development Goals (SDGs). The overriding SDG commitment is to enable a more equitable distribution of essential global resources.^22^ Although inequalities in access to health services between well- and poorly-resourced environments have been documented in considerable detail, there has been limited work on equitable access to scarce resources within the same healthcare system.^2,9,20,21^

Ongoing appraisal of such access is important because healthcare infrastructure and referral pathways may lag changes in population size and distribution and contribute to unintended inequalities.^7,21^ Population growth has been shown to be a key driver of demand for imaging services in the WCP of SA, with the latter demonstrating the second fastest provincial population growth nationally.^7^ Therefore, regular and detailed analyses of both population distribution and resource provision and utilisation are necessary to ensure ongoing equity in healthcare.^21^

Three quarters of the WCP population have no medical insurance and are therefore dependant on public sector health services.^23^ These services are managed along two parallel referral pathways, which link the province’s more peripheral facilities to the three central hospitals, namely Groote Schuur Hospital (GSH), Red Cross War Memorial Children’s Hospital (RCWMCH) and Tygerberg Hospital (TBH).^24^ These pathways have broad geographic constructs and can be conceptualised as ‘western’ and ‘eastern’. GSH and RCWMCH together serve the ‘western’, while TBH serves the ‘eastern’ pathway.^25^

The central hospitals have different bed capacities and patient profiles. GSH, built in 1938, is an adult, maternity, and neonatal centre. It has 893 beds, with 80 dedicated to the neonatal service.^26,27^ RCWMCH was commissioned in 1956 and is an exclusively children’s hospital with 300 beds.^28^ TBH, built in 1976 to accommodate Cape Town’s eastward expansion, provides adult, maternity, paediatric and neonatal services. It has 1386 beds, including 210 for paediatric and 90 for neonatal patients.^29,30^ Each central hospital has a single MR unit. The GSH and TBH MRI units were both commissioned in 2002, while the RCWMCH unit was installed in 2008.

Quantifying MR service utilisation trends aids in defining service pressures and evolving needs. The WCP provides the ideal scenario for an analysis of such utilisation, with three hospitals encompassing different patient profiles across two drainage pathways. This study aimed to analyse public-sector MR utilisation in the WCP of SA.

## Methods

This was a retrospective study of public sector MR utilisation in the WCP of SA in 2013 and 2018. Details of all MR investigations performed on WCP public sector scanners in the respective years were extracted from the official records of the Medical Imaging Services Sub-Directorate (MISSD) within the Directorate of Health Technology of the Western Cape Government Health (WCGH). Data were analysed for the whole province, and by the province’s established ‘western’ and ‘eastern’ referral pathways for central services (see Tables 1a, b and 2a, b).

**TABLE 1a:** Western Cape province drainage populations 2013 and 2018 (total).

GSH & RCWMCH	2013	2018
**Metropolitan**		
Western	513 572	596 805
Southern	543 622	581 179
Klipfontein	395 519	406 071
Mitchells Plain	541 789	594 401
**Rural**		
Garden Route	581 652	609 147
Central Karoo†	64 145	65 215
Saldanha Bay Municipality	103 498	114 709

**Total**	**2 743 797**	**2 967 527**

Note: Groote Schuur Hospital (GSH) and the Red Cross War Memorial Children’s Hospital (RCWMCH) together serve 4 western metropolitan and 2 rural districts. Tygerberg Hospital (TBH) drains 4 eastern metropolitan and 3 rural districts. Exceptions to the drainage patterns are the Saldanha Bay and Laingsburg Municipalities which respectively drain to GSH & RCWMCH and TBH.

GSH, Groote Schuur Hospital; RCWMCH, Red Cross War Memorial Children’s Hospital; TBH, Tygerberg Hospital.

† , Excluding Laingsburg population.

**TABLE 1b:** Western Cape province drainage populations 2013 and 2018 (total).

TBH	2013	2018
**Metropolitan**		
Khayelitsha	410 096	433 502
Eastern	559 317	661 366
Northern	392 461	445 338
Tygerberg	638 168	703 715
**Rural**		
Overberg	262 427	288 836
Winelands district	829 848	909 741
West Coast district†	302 818	333 071
Laingsburg	8546	8964

**Total**	**3 403 681**	**3 784 533**

GSH, Groote Schuur Hospital; RCWMCH, Red Cross War Memorial Children’s Hospital; TBH, Tygerberg Hospital.

† , Excluding Saldanha Bay population.

**TABLE 2a:** Western Cape Province Drainage populations 2013 and 2018 (dependent).

GSH & RCWMCH	2013	2018
**Metropolitan**		
Western	386 720	449 394
Southern	409 347	437 628
Klipfontein	297 826	305 771
Mitchells Plain	407 967	447 584
**Rural**		
Garden route	437 984	458 688
Central karoo†	48 301	49 107
Saldanha Bay Municipality	77 934	86 376

**Total**	**2 066 079**	**2 234 548**

GSH, Groote Schuur Hospital; RCWMCH, Red Cross War Memorial Children’s Hospital.

† , Excluding Laingsburg population.

**TABLE 2b:** Western Cape Province Drainage populations 2013 and 2018 (dependent).

TBH	2013	2018
**Metropolitan**		
Khayelitsha	308 802	326 427
Eastern	421 166	498 009
Northern	295 523	335 340
Tygerberg	480 541	529 897
**Rural**		
Overberg	197 608	217 494
Winelands district	624 876	685 035
West Coast district†	228 022	250 802
Laingsburg	6435	6750

**Total**	**2 562 972**	**2 849 753**

TBH, Tygerberg Hospital.

† , Excluding Saldanha Bay population.

For 2013 and 2018, population figures for the WCP as a whole, and the respective referral pathways, were extracted from the WCP Population Circular (2020), which provides data by year, geographic location, and age.^31^ The analysis was based on 75.3% of the WCP population being dependent on public healthcare^21,23^ and each of the three WCP central hospitals being equipped with a single MR scanner throughout the review period.

Access to, and utilisation of, MR services were expressed as units per million people and studies per 1000 people, respectively, for the whole province and for ‘western’ and ‘eastern’ referral pathways. Sub-analyses defined MR access and utilisation for people aged 0–14 years, and for those older than 14 years.

### Ethical considerations

A retrospective study was performed using data freely available in the public domain.

Study approval was granted by the Stellenbosch University Health Research Ethics Committee (S20/10/296) and Tygerberg Hospital Management. The study was registered on the National Health Research Database (WC_202108_27).

## Results

### Population

#### Analysis for the whole province

Between 2013 and 2018, the WCP population dependent on public health services increased by approximately 455 250 people (8.2%, 4.63 vs 5.08 × 10^6^) (Table 3).^31^

**TABLE 3:** Drainage populations, resource access and utilisation.

Population	2013	2018	Total increase 2013–2018	Total % increase 2013–2018
**Whole province (total population)**	6 147 478	6 752 060	604 582	9.8
**Dependent population**	4 629 051	5 084 301	455 250	9.8
**Dependent population by drainage area:**				
Western pathway	2 066 079	22 345 48	168 469	8.2
Eastern pathway	2 562 972	2 849 753	286 781	11.2
**Dependent population by age and drainage area:**				
**> 14 years**				
Total	3 458 737	3 813 832	355 095	10.3
Western pathway	1 553 697	1 688 042	134 345	8.6
Eastern pathway	1 905 040	2 125 790	220 750	11.6
**0–14 years**				
Total	1 170 314	1 270 469	100 155	8.6
Western pathway	512 382	5465 06	34 124	6.7
Eastern pathway	657 932	723 963	66 031	10.0
**Resource access (MR units/million dependent people)**				
Whole province	0.65	0.59	−0.06	−9.0
**By drainage area:**				
Western pathway	0.97	0.90	−0.07	−7.5
Eastern pathway	0.39	0.35	−0.04	−10.1
**By age and drainage area:**				
**> 14 years**				
Western pathway	0.64	0.59	−0.05	−8.0
Eastern pathway	0.39	0.35	−0.04	−10.1
**0–14 years**				
Western pathway	1.95	1.83	−0.12	−6.2
Eastern pathway	0.39	0.35	−0.04	−10.1
**Resource utilization (total studies)**				
Total cases	10 090	12 848	2758	27.3
**By drainage area:**				
Western pathway	6085	7939	1854	30.4
Eastern pathway	4005	4909	904	22.6
**By age and drainage area:**				
**> 14 years**				
Western pathway	4949	6614	1665	33.6
Eastern pathway	3401	4179	778	22.9
**0–14 years**				
Western pathway	1136	1325	189	16.6
Eastern pathway	604	730	126	20.9
**Resource utilisation (studies/10^3^ dependent people)**				
Whole province	2.18	2.53	0.35	15.9
**By drainage area:**				
Western pathway	2.95	3.55	0.61	20.6
Eastern pathway	1.56	1.72	0.16	10.2
**By age and drainage area:**				
**> 14 years**				
Western pathway	3.19	3.92	0.73	23.0
Eastern pathway	1.79	1.97	0.18	10.1
**0–14 years**				
Western pathway	2.22	2.42	0.21	9.4
Eastern pathway	0.92	1.01	0.09	9.8

#### Analysis by referral pathway

In 2013, 55% of the total WCP dependent population (2.56 × 10^6^ people) lived in the ‘eastern’ referral pathway, exceeding the ‘western’ by approximately 496 000 people. Between 2013 and 2018, ‘eastern’ pathway population growth (286 781) exceeded ‘western’ (168 469) by 70% (118 312/168 469). By the end of the review period, there were approximately 615 000 more dependent people in the ‘eastern’ than the ‘western’ referral pathway.

#### Analysis by age and referral pathway

In 2013, 55% of dependent people older than 14 years (1.91 × 10^6^ people) lived in the ‘eastern’ referral pathway, exceeding the ‘western’ by approximately 350 000 people. Between 2013 and 2018, ‘eastern’ population growth (220 750) exceeded ‘western’ (134 345) by 64% (86 405/134 345). By the end of the review period, there were approximately 438 000 more dependent people of this age in the ‘eastern’ than the ‘western’ referral pathway.

In 2013, 56% of dependent children aged 0–14 years (0.66 × 10^6^) lived in the ‘eastern’ referral pathway, exceeding the ‘western’ by approximately 146 000 children. Between 2013 and 2018, the ‘eastern’ population growth (66 031) exceeded the ‘western’ (34 124) by 94% (31 907/3414). By the end of the review period, there were approximately 177 000 more children aged 0–14 years in the ‘eastern’ than the ‘western’ referral pathway.

### Access to MR services

#### Analysis for the whole province

Given the increasing provincial population and the static MR resources, overall MR access decreased by 9.2% (0.65 vs 0.59 units/10^6^ million people) in the review period.

#### Analysis by referral pathway

‘Western’ access decreased by 7.2% (0.97 vs 0.9 units/10^6^ people) and ‘eastern’ by 10.3% (0.39 vs 0.35 units/10^6^ people).

#### Analysis by age and referral pathway

MR access for the ‘western’ dependent population aged > 14 years and 0–14 years decreased 7.8% (0.64 vs 0.59 units/10^6^ people) and 6.2% (1.95 vs 1.83 units/10^6^ people) respectively, compared to 10.1% (0.39 vs 0.35 units/10^6^ people) for both groups in the ‘eastern’ pathway.

### Overall MR utilisation (total studies)

Provincial MR studies increased 27% overall (10 090 vs 12 848). For patients 0–14 years, there were 17% (1136 vs 1325) and 21% (604 vs 730) increases in the ‘western’ and ‘eastern’ referral pathways respectively, while for older patients the respective increases were 34% (4949 vs 6614) and 23% (3401 vs 4179).

### MR utilisation/10^3^ people

#### Whole province

Utilisation increased 16% (2.18 vs 2.53 studies/10^3^ dependent people).

#### Analysis by referral pathways

‘Western’ and ‘eastern’ pathway utilisation increased 21% (2.95 vs 3.55 studies/10^3^ people) and 10% (1.56 vs 1.72 studies/10^3^ people) respectively. The ‘western’ to ‘eastern’ differential was 1.9:1 in 2013 and 2.1:1 in 2018.

#### Analysis by age

Between 2013 and 2018, MR utilisation for dependent people older than 14 years increased 23% (3.19 vs 3.92) and 10% (1.79 vs 1.97 studies/10^3^ people) in the ‘western’ and ‘eastern’ drainage areas respectively. The ‘western’ to ‘eastern’ differential was 1.8:1 in 2013 and 2.0:1 in 2018.

Between 2013 and 2018, MR utilisation for dependent children aged 0–14 years increased 9.4% (2.22 vs 2.42) and 9.8% (0.92 to 1.01 studies/10^3^ people) in the ‘western’ and ‘eastern’ referral pathways respectively. The ‘western’ to ‘eastern’ differential was 2.4:1 throughout the review period.

## Discussion

To the best of our knowledge, this is the first study to assess equity in access to highly specialised services within the same public healthcare system at provincial level. Therefore, this work makes an important contribution to the discourse on equitable access to essential services, which is at the heart of the UN 2030 Agenda for Sustainable Development.

There are known disparities in healthcare between well-resourced and poorly resourced countries.^5^ Similarly, there are known inequities between healthcare systems (private vs public) and provinces in the same country.^7,19,21,32^ However, to date there has been scant recognition of potential inequalities that can unwittingly exist on the same healthcare platform in a single region, province, or city. This study highlights the key role that the distribution and utilisation of sophisticated radiological equipment can play in providing equity and access to services in this setting. High-end radiological equipment is typically capital and labour intensive, has specific installation and infrastructure requirements, and is registered *by location* with a regulatory authority. It thus provides the ideal yardstick for the measurement of access to highly specialised medical services.

The Western Cape is acknowledged as one of South Africa’s most unequal provinces and is known to have increasing inequality, as measured by the Gini coefficient.^33^ Furthermore, historically, the City of Cape Town (CCT) has had unequal development, with entrenched racial and economic divisions contributed by the combination of peninsular topography and the consequences of apartheid spatial planning.^34,35^ These differences are manifest in the city’s distribution of economic and health indicators. The ‘eastern’ pathway is known to have a higher rate of poverty and a greater burden of communicable diseases.^36^

The key finding of this study is the unintended inequality in access to MR services between the two WCP referral pathways. Review of the history of health services in the WCP provides insights into how this inequality could have evolved over time. It is likely that entrenched referral pathways to the central hospitals have not been modified as the provincial population size and distribution have changed.

Population growth is acknowledged as the major driver of WCP imaging utilisation.^21^ The overall WCP population grew 9.8% in the 6 years of this investigation and was 6.75 million people in 2018. If one considers that the WCP population has grown 68%, from 4.3 million people at the time of commissioning one MR unit in each of the referral pathways in 2002, to a forecasted 7.2 million people in 2022, one can appreciate that current provincial MR resources have not kept pace with population growth.^37,38^

Furthermore, the lesser resourced Eastern referral pathway drains a larger, younger, faster-growing population, with a lower socio-economic index and a higher burden of communicable disease.^33^ It is noteworthy that the effect of population growth on access to finite resources is not limited to MR imaging but can be extrapolated to any centralised, highly specialised health service or public amenity.

A major strength of WCP healthcare is its meticulous, ongoing collection and analysis of data pertaining to the broad range of healthcare services and indicators, including diagnostic imaging. This is underscored by the establishment of the Sub-Directorate Medical Imaging Services in 2007, which is mandated to co-ordinate and assess clinical services, to enhance efficiency and to build capacity within the province’s public sector radiographic services. It also provides advisory support to ensure implementation of, and adherence to, radiation-related legislation and policies.

Additionally, WCP referral pathways to the central services are well defined and carefully considered, taking into consideration transport routes for Emergency Medical Services (EMS), planned patient transport (HealthNet), and public transport. Differential local population growth is influenced by a range of complex socio-economic factors, and manifest over many years. As accurate data on local demographics (at sub-district) has become more available over the years, these are being factored into a system of ‘equity-based resource allocation’.

The ability to collate and integrate accurate data on service utilisation and population growth for the province’s well established referral pathways allows the province to adopt strategies to enhance equity. For example, this work has shown that among patients 0–14 years of age, ‘western’ pathway utilisation (studies/10^3^ people) exceeded ‘eastern’ by a factor 2.4 throughout the review period, and that in 2018, the ‘western’ pathway performed 2.42, compared to the ‘eastern’ 1.01 studies/10^3^ people. Any strategy to enhance equity should thus aim at increasing annual ‘eastern’ utilisation by 1.4 studies per thousand people. Considering the ‘eastern’ population in this age group was 723 963 in 2018, the aim should be to increase annual ‘eastern’ scanning capacity by at least 1000 scans (1.4 × 724). Similarly, among patients older than 14 years, for whom the difference between ‘western’ and ‘eastern’ utilisation was 1.95 scans/10^3^ people in 2018, approximately 6700 additional scans would be required.

Another strategy to enhance equity would be the commissioning of a second scanner in the ‘eastern’ drainage pathway. Its potential impact on access to MR resources for the eastern drainage pathway would be an increase from 0.24 to 0.48 units/10^6^ people. While significant, this is still below the current ‘western’ drainage pathway access of 0.64 MR units/10^6^ people. Other possible interventions to advance equitable access to WCP health resources include the sharing of scanners across drainage pathways and the reconfiguring of population drainage areas. A possible short-term strategy to enhance equity would be further increasing scanning hours on the existing ‘eastern’ pathway scanner.

Strengths of this study are the meticulous Western Cape Provincial medical imaging and population datasets upon which this work is based. The study was limited by the lack of data on the hours of operation of the three MR units. A further limitation is the absence of an analysis on the impact of unequal access on clinical outcomes.

Suggested future work in this field of operational research includes an analysis of the impact of increased ‘eastern’ pathway MR scanning times on utilisation of services and the conduct of similar studies across other imaging modalities, particularly CT and mammography.

## Conclusion

Ensuring equitable services on the same healthcare platform requires ongoing surveillance of resource and population distribution. MR access can serve as a proxy for equity in highly specialised services.
